# The Transformation of Pigment in Fruit Wine, Precise Control of Pigment Formation, and Their Effect on Product Quality

**DOI:** 10.3390/foods14132207

**Published:** 2025-06-23

**Authors:** Xiang Tan, Mengfan Ding, Chen Wang, Linhua Huang, Junying Bai

**Affiliations:** 1 Citrus Research Institute, Southwest University, Chongqing 400700, China; xiangtan@swu.edu.cn (X.T.); huanglh180@swu.edu.cn (L.H.); 2 National Citrus Engineering Research Center, Chongqing 400700, China; wangchen20220307@swu.edu.cn; 3 APC Microbiome Ireland, University College Cork, T12YT20 Cork, Ireland; mding@ucc.ie; 4 College of Food Science, Southwest University, Chongqing 400700, China

**Keywords:** fruit wine, pigment, quality, stability, identification, bioavailability

## Abstract

Global fruit production is excessive, and fruit wine is a significant outcome of fruit processing. The pigment in fruit wine gives it a vibrant color and affects its quality, taste, and marketing. The pigments in fruit wines are commonly divided into three categories: anthocyanins, carotenoids, and chlorophylls. They are naturally synthesized pigments in plants that undergo complex biochemical changes that eventually tend to be stable in mature fruit wine, showing the color properties desired by consumers. Under normal circumstances, pigment molecules are unstable and have isomers, which makes it difficult to accurately identify and control them. In addition, biochemical changes produce a series of chemical derivatives that affect bioavailability and biological functions. This review summarizes the chemical basis, formation process, influencing factors, identification techniques, bioavailability, and bioactivity of fruit wine pigments, providing an important reference for the utilization of fruit resources and the development of high-quality fruit wine products.

## 1. Introduction

Fruit production continues to increase worldwide, and the deep-processing industry faces serious challenges. Fruit wine ranks among the most favored fermented drinks worldwide and has been consumed for thousands of years. Fruits made into fermented wine can extend seasonality, increase economic earnings, and alleviate agricultural and environmental pressures caused by excess fruit production. Almost every raw fruit material can be made into fruit wine; thus, fruit wine is regarded as an important product of fruit processing and possesses huge market application prospects. The quality of fruit wine depends heavily on the raw materials it is made from. The common raw materials are grapes, green plums, berries, apples, and citrus. Wine is the largest category, which accounts for about 80–90% of the total market [1]. Cider is a fruit wine that is, today, the most commercially produced and consumed worldwide and the most popular beverage in many areas [2,3]. Types include green plum wine, which is mainly produced in China, and kiwifruit wine, which is produced and consumed in small amounts worldwide.

Fruit wines contain a wealth of chemical substances like sugars, acids, flavonoids, pigments, and terpenes, which determine their color, taste, and other quality attributes [1]. The color of fruit wine has an important impact on the appearance and marketability of fruit wine products [4]. This is related to the manner in which color is generated by pigment molecules [5]. Color-developing compounds like anthocyanins and carotenoids typically undergo a series of biochemical modifications during the processing and storage of fruit wine [4]. These alterations in structure lead to differences in how pigment compounds remain stable, dissolve, and interact [6]. In addition, fruit wine pigment molecules are mostly flavonoids and carotenoids, which have biological functions and boost the nutritional and health properties of fruit wine. Epidemiological studies have associated pigment-rich diets with several pleiotropic health benefits [7].

Using the keywords such as “fruit wine”, “pigment”, “color”, “pigments”, “wine”, “fruit”, etc., by searching databases like PubMed and Google Scholar from 2000 to 2025, the research progress was established. Based on this, the review discusses the chemical substances forming fruit wine pigments, environmental factors affecting the color appearance of fruit wine, advanced methods for accurate determination of fruit wine pigments, beneficial functions of fruit wine pigments, and their bioavailability. This study helps us understand pigment composition and their dynamic changes in fruit wine production and paves the way for future exploration and advancements of fruit wine production technology.

## 2. Chemical Characteristics of Pigment Molecules in Fruit Wine

### 2.1. Fruit Pigment and Color Presentation

**Figure 1:**
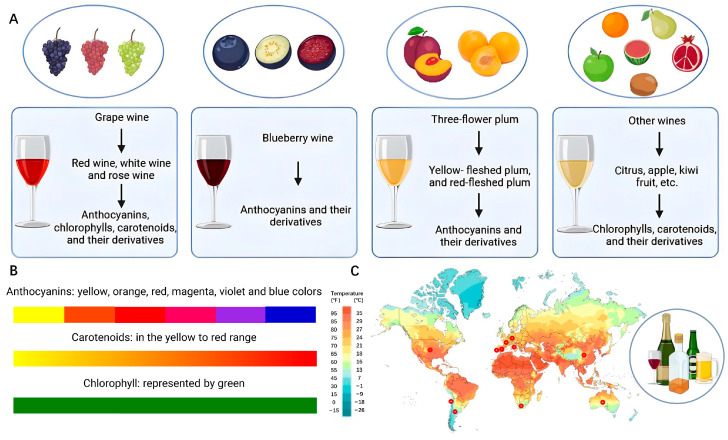
Common fruit wine and their raw materials. (**A**) The characteristics and main color substances of color pigments from common fruit wine and raw fruit categories. (**B**) Color characteristics of common pigments in fruit wine. (**C**) World map and some countries with large fruit wine production.

From the perspective of chemical composition, pigments in fruits are divided into three main categories: anthocyanins, carotenoids, and chlorophyll [8] (Figure 1). Anthocyanins are widely distributed in dark-colored fruits, including red, blue, and purple fruits, such as grapes, blueberries, cherries, and apples [2,7]. Carotenoids such as citrus fruits, tomatoes, and persimmons are mainly found in red, yellow, and orange fruits. Chlorophyll is found in most green fruits, such as melons, kiwifruits, green apples, and watermelons.

Anthocyanins are one type of flavonoid phytopigments [9] that are water-soluble natural colorants [10]. They confer blue, red, orange, magenta, and violet coloration [11]. For eyes, aurones and chalcones exhibit yellow pigmentation, whereas flavones and flavonols are seen as either colorless or faint yellow [11]. In their natural state in fruits, anthocyanins appear as glycosides, where the anthocyanidin molecule (generically known as aglycone) is coupled with sugar [10]. Although hundreds of anthocyanins have been reported, six major basic structures have been identified in fruits: pelargonidins, cyanidins, delphinidins, peonidins, petunidins, and malvidins [12]. The most prevalent species are cyanidins, which have two hydroxyl groups on the B ring, with delphinidins coming next [10]. Notably, because peonidin can originate from cyanidin, and both petunidin and malvidin are sourced from delphinidin, it can also be considered that there are only three primary anthocyanidins: cyanidin, pelargonidin, and delphinidin [11]. These compounds exhibit various colors based on the B-ring substituents, type and number of conjugated sugars, local pH, and coexisting compounds (co-pigments such as flavonols) [12].

The fractionation of anthocyanins in grapes included cyanidin-3-glucoside, delphinidin-3-glucoside, malvidin-3-cumarylglucoside, malvidin-3-acetylglucoside, malvidin-3-glucoside, peonidin-3-acetylglucoside, peonidin-3-glucoside, peonidin-3-cumarylglucoside, and petunidin-3-glucoside [13]. Cyanidins are abundantly distributed, especially in red berries, such as blueberries, sweet cherries, mulberries, bayberries, and black chokeberries [14]. The blueberries contain only pelargonidin. Pelargonidin 3-glucoside gives strawberries most of their red color, although strawberries also contain cyanidin 3-glucoside. Pomegranate juices are violet due to delphinidin being the primary anthocyanidin, whereas those with pelargonidin as the main anthocyanidin are scarlet.

Carotenoids are best known as crucial natural pigments in the yellow to red range in the peel of mature fruits, such as citrus fruits, that determine their color [13]. Structurally, carotenoids belong to the terpenoid category and are usually condensed from isoprene units [15]. The specific number of carbon atoms and chain length of the carotenoids (C30–C50) depend on the number of C5 isoprene units [7]. The Carotenoid Database (http://carotenoiddb.jp, accessed on 17 February 2018) provides data on the chemical structures of 1158 carotenoids identified in 691 organisms [7]. The conjugated double-bond system of carotenoids is responsible for their color (chromophore), shape, reactivity, and photochemical properties [7]. Carotenoids are divided into two main classes based on their chemical and nutritional characteristics: carotenes and xanthophylls [16]. Carotenes, which include lycopene, α-carotene, and β-carotene, as well as other less-studied classes, like γ-carotene, δ-carotene, phytoene, zeta-carotene, phytofluene, and neurosporene, are unoxygenated terpenes (composed solely of carbon and hydrogen atoms) [17]. Meanwhile, the xanthophyll category, which mainly comprises lutein, zeaxanthin, β-cryptoxanthin, canthaxanthin, and violaxanthin, is oxygenated (containing oxygen) [17,18]. Oxygenated radicals generally in xanthophylls include hydroxyl (lutein and zeaxanthin), epoxide (violaxanthin and neoxanthin), and carbonyl (canthaxanthin and capsanthin) groups [7]. Numerous carotenoids have chiral centers within their molecular structures; thus, optical isomers such as zeaxanthin can occur [7]. In fruits, lycopene, α-carotene, and β-carotene are primarily present as all-trans isomers [19].

Significant amounts of carotenoids are found in fruits such as citrus, orange, pomegranate, ferocissimum fruits, guava, and Thai gac fruit (Momordica cochinchinensis Spreng) [20]. A previous report has suggested five unique carotenoid patterns associated with color [7]: (1) acyclic carotene lycopene related to red color, as in tomatoes; (2) β-carotene and/or its hydroxyl derivates β-cryptoxanthin and zeaxanthin in relation to orange color; (3) α-carotene and/or its hydroxyl derivates related to yellow–orange color, especially lutein; (4) carotenoid epoxides (yellow color); and (5) unique carotenoids related to yellow, red, or orange color, e.g., capsorubin and capsanthin. Xanthophylls esterified with fatty acids are the primary carotenoids found in most fruits [7]. Orange fruits accumulate β-carotene and an elevated level of xanthophylls, notably β,β-xanthophylls [7]. In all the grape berries, lutein and β-carotene were the most prevalent carotenoids, making up 85% of the total carotenoids in grape [21]. The leftover carotenoids include xanthophylls like neochrome, violaxanthin, neoxanthin, luteoxanthin, flavoxanthin, lutein-5,6-epoxide, and zeaxanthin, and cis isomers of lutein and β-carotene [19]. The key sources of lycopene are tomatoes and derived products, as well as watermelon, papaya, guava, and pink grapefruit [5,21]. β-Cryptoxanthin is widely present at low concentrations in fruits, but it is a major carotenoid in ripe orange and red mangos, papayas, mandarins, oranges, and persimmons [7]. During storage, the levels of β-cryptoxanthin and 9-cis-violaxanthin (β, β-xanthophyll) kept rising, while the amounts of lutein, β-carotene, and all-trans-violaxanthin steadily declined [15]. Rich synthesis of carotenoid derivatives in the β,β-branch might be responsible for the β-carotene loss [15]. It is noteworthy that several carotenoids, including lycopene and β-carotene, are approved as food colorants [22]. Lycopene’s acceptable daily intake as an additive is set at 0.5 mg kg^−1^ body weight per day [22].

Chlorophylls are crucial pigments responsible for the green coloration of fruits, and the color pigments of green fruits and immature fruit peels mainly consist of green chlorophylls [15]. Chlorophyll is a complex molecule comprising four pyrrole rings, a magnesium atom, and a long phytol chain [21]. The chlorophyll content in fruits and peel is generally expressed either as total chlorophyll, chlorophyll a, or chlorophyll b [15]. Structurally, chlorophyll b has one more carbonyl group than chlorophyll a and is, therefore, more soluble in polar solvents. Common fruits that produce chlorophyll include green apples, grapes, pears, kiwis, and immature oranges. In ripe green fruits, the chlorophyll content is found to decrease during storage [15].

Multiple pigmentation molecules may be present in the same fruit. At different stages of fruit ripening, the change in color is accompanied by the mutual conversion of different pigment molecules. Citrus fruits experience a natural process called degreening, where the peel color shifts from green to yellow or orange as they mature [15]. Chlorophyll degradation and carotenoid synthesis are a result of the color break from the “unripe” green to “ripe” yellow/orange [15]. In a previous report on apples, cyanidin was found as the primary anthocyanin in the red skin, delphinidin was not detected, and quercetin glycosides were detected in both green and red skin [23]. The color of the exocarp (commonly called the peel) of the white grape variety is mainly due to the presence of light-yellow-colored polyphenols, such as flavonols, which are dominated by quercetin-type flavonols and carotenoids [24]. Knowledge regarding the concentration of carotenoids and chlorophyll in the berry peels of these white varieties is scarce; thus, fruit ripening is based only on visual color, sugar, and/or acid content [24]. Many fruit varieties contain different pigments in their flowers. The yellow-flowered plants produce carotenoids, whereas the orange-flowered plants produce pelargonidin-related anthocyanins [11].

### 2.2. The Characteristics of Color Pigments from Common Fruit Wine Categories

Pigments in fruits eventually exist in fruit wine after undergoing various biochemical transformations in the winemaking process, which gives fruit wine its unique color properties. Common fruit wines include many different categories, such as wine from red and white grapes and fruit wines made from black mulberry, quince, blackberry, apple, citrus, apricot, red raspberry, melon, sour cherry, bilberry, and strawberry [22,25] (Figure 1).

Wine is deeply loved by consumers in many countries. The United States, France, Italy, Germany, and China are the five countries that consume the most wine worldwide, at 13.3%, 11.2%, 9.4%, 8.1%, and 7.2%, respectively [26]. Color is a crucial aspect of the sensory perception of wine and is also the most direct quality characteristic [27]. Wine pigments originate from a variety of ingredients and sources, but all originate from grapes. Flavonols, anthocyanins, and their derivatives are the most important pigments in wine. Young wine mainly contains primary anthocyanins, which give red grape varieties their color, whereas new anthocyanin derivatives that are important in older wine are formed during fermentation and aging. These pigments can be either monomeric or polymerized. Based on their structure, they are often classified as pyrananthocyanins or non-pyrananthocyanins (in the latter case, also known as polymer pigments). The concentration of monomeric anthocyanins in wine depends largely on the grape variety, winemaking technique, and aging time [28]. These anthocyanins are degraded and absorbed by yeast during alcohol fermentation and then further transformed into anthocyanin derivatives in the presence of specific metabolites and phenolic acids during aging and storage [29]. Anthocyanin derivatives are produced by reactions between anthocyanins and other substances and are essential for the long-term color performance of wine [28]. Owing to anthocyanin degradation and other reactions, the average anthocyanin content of young wine (aged < 6 months without oak contact) is 400 mg/L, whereas that of old wine (aged 2 years in contact with oak) is 90 mg/L. With longer aging times (>10 years), only a small amount of anthocyanins (if any) are available, and the color is mainly attributable to anthocyanin derivatives and pigment polymers formed during winemaking and aging. Wines with the highest concentrations of anthocyanins do not necessarily have the highest color intensity [30].

Other components supporting the color of wine include chlorophylls and their precursors, catabolites, quinones, carotenoids, and lipids, which play important roles in the integrated sensory properties of grapes and wine [31]. The skins of ripe red grape berries have chloroplasts, and the number of chloroplasts (and the amount of chlorophyll) in the pigmented skin is equivalent to their abundance at the green stage [31]. Among the samples analyzed, the monovarietal *Vitis vinifera* L. cultivar wine was among the five most important Douro varieties, Tinta Roriz contained the highest levels of carotenoids, and Touriga Franca contained the lowest [32]. Chardonnay and Merlot wines have higher concentrations of norisoprenoids, which originate from the direct degradation of carotenoid molecules such as β-carotene, lutein, neoxanthin, and violaxanthin [33]. Additionally, in Chardonnay wines, a higher level of β-damascenone (a neoxanthin derivative) is observed than β-ionone one (a β-carotene derivative) [33].

Blueberry wine is a novel fruit wine with good taste and rich nutrition; however, color change and anthocyanin content readily decrease during the production process. Anthocyanins are the main source of color and nutrients in blueberry wines. However, during fermentation, anthocyanins are exposed to various factors that contribute to their decomposition. Studies have reported that anthocyanins are less stable at room temperature than during cold storage [34]. Anthocyanins are sensitive to vitamin C, oxidants, reducers, and preservatives at room temperature. The anthocyanins in blueberry wine include delphinidin glucoside, morning glory galactoside, cornflower glucoside, and malvidin galactoside. Malvidin is purple–red, and its increased content can contribute to the darkening of blueberry wine color.

Three-flower plums, including yellow- and red-fleshed plums, are widely used as raw materials for fruit wine. The skin and flesh of ripe fruits gradually change from green to purplish–red, and anthocyanins accumulate continuously during this process [35]. The typical pigments identified in *Prunus triflorus* are anthocyanins and pelargonidins, both of which belong to anthocyanins [35]. Proanthocyanidins B1 and B2 were present in all plums, whereas cyanidin-3-O-glucoside was the dominant anthocyanin in the South African plums [29]. The main disadvantages of using plums to make fruit wine are their relatively poor anthocyanin content and the more volatile variety of anthocyanins compared with grapes, blueberries, and other anthocyanin-rich fruits [29]. After the fermentation and aging of plum wine, there is a significant difference in the observed color attenuation effect, which directly threatens the sensory quality of the plum wine and greatly reduces its shelf life. Therefore, it is essential to protect intrinsic and restrictive anthocyanins for better color appearance.

## 3. Factors Affecting Pigment Formation and Stabilization in Fruit Wine

### 3.1. Key Steps in the Fruit Winemaking Process

The pigments in fruit wine are not exactly the same as the pigments in raw fruit because the production process involves a set of complex enological steps that might affect them [30]. According to previous studies, the key steps of the winemaking process can be summarized as follows: crushing, fermentation, stabilization, and aging/maturation [36]. First, intact fruits are crushed or squeezed into juice as a raw material. Second, the raw materials are modified by adding potassium metabisulfite, pectolytic enzymes, and sugar. Third, fermentation is performed by inoculation with active dry yeast powder. Fourth, when the fermentation liquid’s residual sugar level drops under 2 g/L, the liquid is racked, potassium metabisulfite is added, and the bottle is sealed for aging and clarification over 10–14 days to obtain fruit wine.

**Figure 2:**
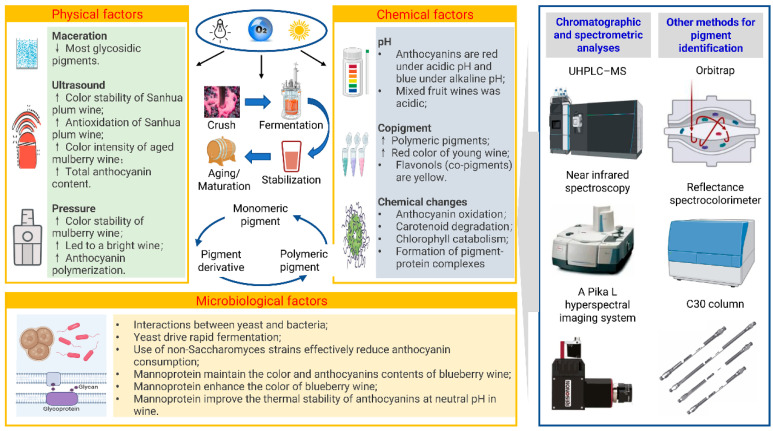
The transformation and identification of pigment molecules during different periods of fruit wine fermentation and their effects on fruit wine quality. The left side shows the factors that affect the pigment change during fermentation, and the right side shows some advanced methods currently used for the accurate identification of pigment molecules.

The fermentation process involves microbial biochemical action on the pigments. For example, during the alcoholic fermentation of wine brewing, pyranoanthocyanins and polymeric pigments are produced [37]. Optimal nonthermal maturation techniques, including ultrasound, high-pressure, and manosonic maturation, guarantee microbial safety and better polyphenolic pigment preservation [38]. Other steps in fruit wine production also have important effects on the color and sensory quality of fruit wine [38]. Therefore, the factors affecting the formation and stability of pigments in fruit wine include physical, chemical, and microbial factors (Figure 2). For example, anthocyanin stability is impacted by pH, light, and temperature and also depends on the B-ring within their framework and the existence of methoxyl or hydroxyl groups [39], which could be associated with fatty acids, proteins, sugars, or other substrates that can change their physical and chemical properties and influence their biological roles [7].

### 3.2. Physical Factors

**Cultivar and preharvest environment.** The pigment content in fruits and fruit wine is affected by the cultivar and preharvest environmental status [13]. The concentration of anthocyanins in fruits is associated with color intensity and is related to differences in altitude, region, and environmental factors, such as temperature, exposure to light, and water availability [40]. Because these fruit wines were prepared using the same winemaking method, these differences in color intensity can be clearly attributed to the difference in fruit pigment content caused by environmental differences. Severe drought in the soil may increase phenolic compounds, particularly anthocyanins, in grape skin [41]. Generally, grapes from hot climates exhibited the highest carotenoid levels, yet mature grapes exposed to sunlight showed lower carotenoid concentrations than those grown in the shade [33]. The cultivar, viticultivar region, exposure to sunlight, and ripening stage affected carotenoid levels in grapes [33]. A detailed observation revealed a higher level of β-damascenone (a neoxanthin derivative) in Chardonnay wines than β-ionone one (a β-carotene derivative) [33].

**Time, temperature, humidity, and radiation.** High temperatures and solar radiation induce a transition from hydroxylated (cyanidin and delphinidin) to methoxylated derivatives of anthocyanins (malvidin, peonidin, and petunidin) [13]. Heat treatment increases the cavities on the most exposed surfaces of French oak wood fragments and the materials’ maximum humidity by 20%, which leads to an increase in the yellow color of wine [42]. The total carotenoid levels during the processing and storage periods and their kinetics of change can provide a useful tool for maintaining and improving the quality of wines [33]. Aged Port wines displayed greater beta-carotene to lutein ratios than the new Port wines [32]. Conversely, young Port wines had greater amounts of total carotenoids and chlorophyll-like substances than aged Ports, with lutein and beta-carotene being the primary carotenoids [32]. A forced-aging study on grapes and Port wines implied that lutein is more responsive to temperature variations compared to beta-carotene [32]. The degradation rates of chlorophyll derivatives were faster than those of carotene and lutein [32].

**Ultrasound**. Ultrasonic treatment generally occurs during the aging and maturation of fruit wine. The physical acceleration of aging under ultrasonic treatment is conducive to the maintenance of anthocyanins in blueberry wine [43]. Enhanced color stability and antioxidant activity of Sanhua plum wine were also observed under cyclic ultrasound because ultrasonic treatment inhibited the activity of polyphenol oxidase, which is related to the reduction of anthocyanin degradation [43]. Therefore, ultrasonic technology can be used after fermentation to improve Sanhua plum wine color [43]. The chromatic indices of non-thermally aged mulberry wine correlated with flavonols and anthocyanins [38]. Ultrasonic treatment increased the color intensity of aged mulberry wine [38]. This is explained by the fact that ultrasonication may partially inhibit polyphenol oxidase and peroxidase associated with enzymatic browning, which is known to be involved in monomeric anthocyanin degradation and leads to a higher monomeric anthocyanin content recorded in aged ultrasonicated mulberry wine than in the control [38]. Sonication influences the rates of isomerization, polymerization, and depolymerization, which may affect total anthocyanin content [44]. Weak ultrasonic waves promote an increase in total anthocyanin content by influencing the rate of polymerization reactions during wine maturation [44].

**Maceration**. After 90 days of maceration, three anthocyanins in bayberry-soaked wine significantly increased in abundance, including frigarin, aurantinidin, and peonidin-3-O-galactoside, and six anthocyanins showed a notable reduction in their abundance, including cyanidin-3-O-sophoroside, cyanidin-3-O-rhamnoside, and cyanidin 3-O-galactoside-5-O-glucoside [14]. Throughout maceration, bayberry wine experiences a clear decline in most glycosidic pigments. Differential anthocyanin analyses conducted across various maceration periods revealed different fates of the components in bayberry wine, with a marked drop in the majority of glycosidic anthocyanins. According to correlation analysis, the red hue of bayberry wine was predominantly linked to cyanidin-3-O-glucoside, delphinidin-3-O-arabinoside, cyanidin-3-O-rhamnoside, and delphinidin-3-O-galactoside. This helped us understand the composition and dynamic changes in anthocyanins in bayberry wine [1].

**Pressure.** High-pressure maturation enhances the color stability of mulberry wine [38]. This is a result of condensation reactions caused by pressure, which affects the molecules associated with anthocyanin degradation and/or facilitates the production of new pigments, resulting in a decline in monomeric anthocyanins and flavonols [38]. In contrast to ultrasonication, the decrease in the total flavonol index by high-pressure treatment may be a result of the chemical oxidation caused by pressure [38]. In addition, pressurization may increase the polymerization of flavonols and anthocyanins, leading to insoluble mulberry wine components with high degrees of polymerization that are predominantly present in the deposits [38]. The increase in color tonality during pressurization is probably attributed to the generation of yellow pigments like xanthylium salts by the oxidative conversion of flavonols and/or the direct condensation of anthocyanins and flavonols [38]. Anthocyanin polymerization enhances lightness [38]. This suggests that pressurization led to bright mulberry wine, whereas sonication resulted in dark mulberry wine [38].

**Others.** Carotenoids are unstable natural pigments owing to their double-bonded conjugated configurations [21]. These compounds are present in natural sources, mainly in the trans configuration, and isomerize to the cis configuration after contact with acids, heat treatment, and exposure to light [21]. Recent findings revealed that red light exposure can trigger the accumulation of β-cryptoxanthin [15]. Manosonication results in a mulberry wine with a low blue tone [38]. Polymerization, depolymerization, condensation, and isomerization reactions occur during sonication, and pressurization may affect mulberry wine anthocyanins [38]. This sensitivity is also observed in chlorophyll, with Chl b generally being more stable than Chl a [45]. All physical factors are concluded and compared in Table 1.

### 3.3. Chemical Factors

**pH.** Acidity is a key chemical factor affecting the formation and stabilization of pigments. Anthocyanins are responsible for the highest intensity of red color in fruit wine, but they can present other colors depending on pH variation [30]. Anthocyanins are red in acidic environments and blue in neutral in alkaline environments [36,46]. At a basic pH, the instability of anthocyanins leads them to decompose into dark brown oxidized components [39]. In environments with high acidity, anthocyanins take on the form of flavylium ions, appear red, and are quite stable. In contrast, under conditions of weak acidity or neutrality, anthocyanins interact with water, resulting in the formation of unstable, colorless pseudo-bases [10]. The pH of mixed-fruit wines is generally acidic throughout the fermentation period [47]. The pH of fruit wine made from pawpaw, banana, and watermelon ranges from 2.5 ± 0.01 to 3.8 ± 0.01 [47]. A lower pH would generate an improved perception of sugar/acid balance, an enhanced quality of red color, and a reduction in color and aroma evolution by oxidation [30].

**Gas.** Photosynthetic pigments, like chlorophylls and carotenoids, are highly prone to degradation when exposed to oxygen [21]. Therefore, winemaking procedures can influence the quantitative and qualitative profiles of carotenoids in grapes [21]. In addition, the oxidation of phenolic substances produces new pigment molecules, resulting in fruit wine browning [28]. However, differences in color intensity and juice hue were unlikely to result from oxidation since SO_2_ levels consistently exceeded the set limit to avoid oxidation-related issues in juice and fruit wine [48]. Carbon dioxide treatment also affects carotenoid and chlorophyll levels in fruits and fruit wine. Elevated CO_2_ levels significantly facilitated the decomposition of chlorophyll a and b, as well as total chlorophyll content [15]. However, the effects on carotenoids are double-sided. Higher CO_2_ concentrations led to a decrease in total carotenoids but hastened the accumulation of orange β-cryptoxanthin; conversely, a significant reduction was found in lutein, β-carotene, 9-cis-violaxanthin, and all-trans-violaxanthin contents in the CO_2_-treated group [15]. Notably, the level of 9-cis-violaxanthin continuously increased in the control group but decreased progressively in the CO_2_-treated group during storage, which caused a decreased total carotenoid content in the CO_2_-treated group [15].

**Co-pigment.** Co-pigmentation is a solution phenomenon involving spontaneous non-covalent interactions between pigments and co-pigmented molecules (i.e., primary non-anthocyanin phenolics) [34]. Generally, it produces a hyperchromatic effect (more intense color) and a deep color shift (purple hue) in fruit wine [34]. In young wine, an estimated 30–50% of the color intensity is due to pigmentation, but these effects are not permanent and gradually diminish due to the degradation of pigments and parapigments. With age, polymer pigments and pyrananthocyanins play increasingly important roles in the color of wine. Polymeric pigments (compounds that do not bleach in the presence of SO_2_) are generated by condensation reactions between anthocyanins and other phenolic compounds and begin very early in the fermentation process [27]. These polymerization reactions are normal in *Vitis vinifera* wines and help stabilize the wine [27]. Understanding the composition and changes in the colorants of fruit wine is of great significance for improving the quality of fruit wine. Flavonols are the best co-pigmentation factors involved in the formation of stable polymeric pigments during the maturation of aged mulberry wines [38]. Flavonols (co-pigments), present in copy form or in combination with sugars (i.e., glycosides), are yellow pigments [11]. Many berries are rich in flavonols [27]. The effectiveness of flavonols on fruit wine color depends on the co-pigmentation effect of anthocyanins. Anthocyanins lead to the red color of fruit wine, whereas flavonols and flavan-3-ols can strengthen the red color of young fruit wine through the co-pigmentation process [38]. Regarding color composition, flavonoids, anthocyanins, and tannins were the phytochemical groups most closely connected to the chromatic index, with rutin, morin, myricetin, quercetin, cyanidin-3-O-rutinoside, and cyanidin-3-O-glucoside, as the primary agents responsible for the color alterations [38]. These findings and applications potentially strengthen the co-pigmentation between non-flavonoid polyphenols, gallic acid, and malvidin-3-O-glucoside in wine (stabilizing role) [13].

**Chemical changes.** Chemical changes in fruit wine pigments include hydroxylation, methylation, polymerization, and cleavage. The hydroxyl substitution on the B-ring conjugating with the benzene ring intensifies the yellow hue and weakens anthocyanidin stability [49]. An increase in the number of hydroxyl groups on the B-ring imparts a bluer color to the anthocyanins derived from the anthocyanidin, but methylation of the 3′ or 5′-hydroxyl group gives them a slightly redder appearance [11]. Dihydroxylated anthocyanins, such as cyanidin and peonidin, appear red, whereas trihydroxylated anthocyanins, such as malvidin, delphinidin, and petunidin, appear blue and purple [13]. Anthocyanins are responsible for the color of young fruit wine, which evolves during storage from the bluish–red of young fruit wine toward the yellow–orange tones of matured fruit wine, primarily due to anthocyanin oxidation [27]. Carotenoid degradation yields various derivatives. For example, the direct decomposition of carotenoid molecules lutein, β-carotene, violaxanthin, and neoxanthin form C13 norisoprenoids [21]. Each derivative is specific to the initial pigment and is highly flavorant; therefore, it has a noticeable effect on wine color and flavor [33]. The mechanisms underlying carotenoid decomposition include enzymatic processes, autoxidation, and thermal decomposition [33]. It has been recently reported that β-damascenone can be directly generated from 9′-cis-neoxanthin via chemical oxidation when subjected to high heat [33]. β-Ionone could derive from β-carotene oxidation, while 3-hydroxy-α-ionone and 3-oxo-α-ionol could derive from lutein cleavage [33]. The degradation of these compounds may result in color attenuation and the formation of aroma molecules, which can affect the wine flavor [33]. However, some carotenoids can form complexes with water-soluble proteins (carotenoproteins), which appear to stabilize carotenoids [7]. Chlorophyll catabolism involves chlorophyll binding, a/b transformations, and degradation [15]. It has been reported that chlorophyll a/b-binding proteins can bind to pigments and thus prevent the degradation of chlorophyll; however, breaking down pigment–protein complexes is a key early step in the chlorophyll degradation pathway [15].

**Others.** Soluble sugars increase after a decoupling of sugar and anthocyanin, which leads to a poor color in wine [50]. The more soluble sugars remain in unfiltered wine (>3 g/L), the more acetaldehyde is produced [51], which helps enhance the accumulation of pyranoanthocyanins in wine [52]. The addition of fining agents, such as chitosan, a natural cationic flocculant, during the clarification process, lightens the color of citrus wine liquor owing to pigment adsorption [36]. Moreover, gelatin and agar can be combined with phenolic substances to lighten citrus wines by adsorbing pigments [36]. Mannoproteins maintain the anthocyanin content and color of blueberry wine [34]. Although Zn is involved in the biosynthesis of chlorophyll, colorimetric analyses do not show visible changes, indicating that Zn treatments do not influence the relative proportions of chlorophylls and carotenoids that determine the final white grape berry color or the color of the fruit wine, which is an important aspect for consumers [41]. All chemical factors are concluded and compared in Table 2.

### 3.4. Microbiological Factors

The nutritional and sensory qualities of fruit wine do not depend solely on the actions of a single microorganism [54]. Various microorganisms, including yeasts, bacteria, and mold, are often involved in fruit wine fermentation. The interactions between these microorganisms (e.g., yeast–yeast and yeast–bacteria) also play a crucial role in the quality of fruit wine. The production process of fruit wine involves the action of microorganisms to produce a satisfactory color, flavor, appearance, and texture of finished fruit wine [50,51]. Although studies have reported that, in some cases, yeast is a spoilage species or opportunistic pathogen during fermentation, which can cause adverse effects [55], fruit wine can be rapidly fermented by yeast-like fungi, such as *Saccharomyces*, *Pichia*, and *Candida*, which initiated the fermentation process and promoted the biochemical transformation of pigment molecules [48,49]. In addition, there is sufficient evidence that the preferential selection and use of non-*Saccharomyces* strains can effectively reduce anthocyanin consumption during the fermentation stage. Bacterial genera, including *Acetobacter*, *Curtobacterium*, and *Lactobacillus*, are the most highly represented and common acid-producing bacteria and are associated with the color of fruit wine [48,51]. Grey mold like *Botrytis cinerea* increases the concentration of gluconic acid and decreases and maintains the balance of acidity, which in turn affects the stability of pigment in fruit wine [56].

Mannoprotein derived from the cell wall of *Saccharomyces cerevisiae* used for fermentation is an important polysaccharide present in fruit wine [34]. Mannoproteins maintain the color and anthocyanin content of blueberry wine during the main fermentation and post-fermentation stages [34]. Delphinidin is blue, and mannoprotein can maintain delphinidin content to enhance the color of blueberry wine. The effect of mannoprotein on blueberry wine increased as mannoprotein concentration increased [34]. Moreover, mannoproteins stabilize tannins in wine and improve the thermal stability of anthocyanins at neutral pH [34]. The factors regarding microorganisms are concluded and compared in Table 3.

## 4. Pigment Identification for Premise Control of Fruit Wine Color

The pigment compounds in fruit wine were investigated mainly by chromatographic and spectrometric analyses, which were performed by matching the chromatographic and spectrometric properties with standards [33] (Figure 2). High-performance liquid chromatography (HPLC) and mass spectrometry (MS) are the most common methods applied in pigment analyses [23]. The application of high-resolution hybrid MS coupled with HPLC imports precise detection of pigment profiles in food specimens [20]. HPLC-DAD and HPLC-DAD-MS (ESP+) analyses were used to investigate the carotenoid- and chlorophyll-derived components in grapes and Port wines [32]. In Port wines, 19 compounds with carotenoid- or chlorophyll-like structures were present, and in grapes, a total of 13 carotenoid- and chlorophyll-derived components were formally found, and three were identified for the first time [32]. By using HPLC-DAD-MS (ESI+), zeaxanthin and lutein (a pair of structural isomers), as well as lutein and β-carotene (the isomers), were resolved successfully and identified efficiently [33]. Advanced MS technologies like ultra-high-performance liquid chromatography (UHPLC)-MS and data-independent MS (MSE) allow the precise quantification of hormones derived from carotenoids and apocarotenoids [20]. Linked scanning mass spectrometry helps to accurately identify carotenoid components despite contamination being present [20]. Ambient MS techniques, such as desorption electrospray ionization (DESI) and direct analysis in real time (DART), have been used for high-throughput analysis [23]. Using the PESI/MS/MS method, various anthocyanins that were not found in earlier studies could be detected in many red cultivars [23]. Moreover, the pigmented polymer concentration in fruit wine can be determined by gel permeation chromatography (GPC) [57]. These advanced analytical methods clarify the chemical nature of pigments and help precisely control their production process.

Recently, near-infrared spectroscopy (NIRS) and mid-infrared spectroscopy (MIRS), in combination with chemometric analysis, have also been applied to quantify pigment and polyphenol contents [58]. A portable sensor (Multiplex) using a nondestructive fluorescence-based technique was applied to evaluate anthocyanins, chlorophyll, flavonols, and chlorophyll in grapes, which enabled the examination of the spatial heterogeneity of anthocyanin content [59]. Carotenoid compounds in wine grapes grown in the Apulian region were investigated by spectrometric and chromatographic analysis, and cis-isomers of lutein and beta-carotene (9Z-beta-carotene and 9Z, 9′Z-lutein) and 5,6-epoxyxanthophylls were detected: violaxanthin, 9′Z-neoxanthin, and 5,6-epoxylutein [33]. A Pika L hyperspectral imaging system (400–1000 nm) was applied to acquire hyperspectral image information on chlorophyll levels and chlorophyll fluorescence parameters from grape leaves [60]. This system is also expected to be applied in the detection of fruit wine chlorophyll.

In addition, a global untargeted metabolomics technique uncovered 20 distinct anthocyanins in bayberry wine, including cyanidin, peonidin, delphinidin, and malvidin [14]. A color chart and reflectance spectrocolorimeter were applied in combination with analyses of total carotenoids and chlorophyll in all three varieties [24]. A strict correlation was found between the hue angle (measured using a color chart or spectrocolorimeter) and chlorophyll disappearance [24]. Total carotenoid and chlorophyll contents were analyzed, as well as berry peel color changes, via a colorimetric chart and a spectrocolorimeter [24]. Because a single method has certain limitations, there are many cases in which analysis methods are combined. For example, HPLC combined with MS and UV, and DAD visible spectrophotometry, supercritical fluid chromatography, and thin-layer chromatography (TLC) offer reliable techniques for accurately identifying and quantifying pigment molecules, even if similar components are present [20].

Pigment molecules, such as anthocyanins and carotenoids, are highly susceptible to a range of physicochemical attacks, such as light, temperature, and oxygen, which may have a profound effect on their structural and configuration properties [61]. Hence, it is crucial that optimal sample preparation before analysis and analytical steps, such as solid phase extraction, are applied to guarantee that these properties of the single chromophore are intact, as they are essential to effective pigment detection and quantitative analyses [61]. Another important factor is the potential disruption caused by intricate biological matrices, which can hinder pigment detection and require advanced purification and isolation techniques [20]. Currently, different technologies are available for overcoming this interference in pigment analyses. Anthocyanins hydrolyzed into anthocyanidins are usually integral because of the difficulty in gaining anthocyanin standards, which is typically achieved by refluxing HCl and alkaline solutions. Pigments were extracted by an array of solvents, with chloroform as the extraction solvent for pigment isolation [61]. It provides a 10-fold higher carotenoid extraction efficiency and eliminates unwanted UV disruption during online photodiode array (PDA) analysis following HPLC [61]. Online supercritical fluid extraction combined with supercritical fluid chromatography and mass spectrometry imparts the efficient and accurate determination of carotenoids and their cleavage products [20]. A C30 column developed recently gave the highest separation selectivity for a range of carotenoids, which, combined with a binary mobile solvent system, was used for the baseline separation of eight major carotenoids and two chlorophylls (a and b) within 18 min [20,61]. Other extraction methods, such as SPE, have also been used and are occasionally accompanied by MS to help identify glycosides [1]. Operations that protect against light, heat, and oxygen are crucial for preventing degradation and maintaining integrity during isolation and identification [20].

## 5. Effects of Fruit Wine Pigment on Human Health

**Figure 3:**
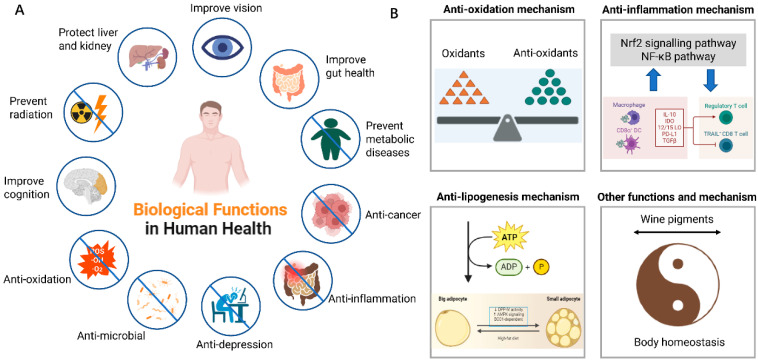
An overview of biological activities and action mechanisms of fruit wine pigments. (**A**) The effect of fruit wine pigments on human health. (**B**) Potential mechanism of fruit wine pigments to improve body homeostasis.

Because of its rich polyphenol and pigment contents, fruit wine has been widely recognized for its impact on human health (Figure 3). The protective effects of wine on cardiovascular health are well known because of its rich polyphenol content [62]. Citrus wine, one of the most abundant dietary sources of flavonoids and carotenoids, has a wide range of beneficial health effects [15]. Sanhua plum wine brewed from Sanhua plums contains amino acids, mineral elements, a variety of polyphenols, alkaloids, and polysaccharides and thus has excellent biological activities [43].

Anthocyanins exhibit strong antioxidant and anti-inflammatory activities. Additionally, the effect of anthocyanins on improving metabolic diseases, brain function, and other aspects has been sufficiently reported, although robust clinical evidence supporting the health benefits of anthocyanins in humans remains insufficient [10]. The function of carotenoids, especially lutein, in improving vision is widely recognized, and the amount of dietary carotenoid intake and circulating levels have been associated with a reduced incidence of obesity, inflammation, impaired vision quality, degenerative chronic diseases, and even certain types of cancer [63]. Mechanically speaking, carotenoids and apo-carotenoids probably could target transcription factors, i.e., NF-κB, PPARγ, and RAR/RXRs [63]. The bioactivity of chlorophyll is attributed to its ability to act as an antioxidant, antimutagen, and anticarcinogen [6]. Chlorophyll derivatives that undergo structural modifications exhibit enhanced bioactivity compared to native chlorophylls [6]. These reports highlight the intricate and multifaceted functionality of pigment molecules within the human body, demonstrating their involvement in essential cellular processes and their potential as therapeutic agents [20].

### 5.1. Anti-Oxidation Capacities

Pigment molecules and pigment-rich products have a strong ability to clear free radicals and inhibit lipid peroxidation. Fruit wine produced from strawberries and drupe fruits showed an anti-oxidative stress ability in rat synaptosomes by activating anti-oxidative enzymes and decreasing malondialdehyde (MDA) levels due to the existence of biologically active compounds such as polyphenols [64]. Wine consumption is associated with reduced oxidative stress [55,65]. Ultrasonicated mulberry wine possessed more antiradical properties, followed by manosonicated and pressurized mulberry wine [44]. Mulberry wine is usually compared to wine in terms of its positive health benefits because of its documented high antioxidant effects associated with the presence of phenolic acids, flavonoids, and anthocyanin compounds [44]. Berry wines possess antioxidant activity in the oxidation of methyl linoleate, and fruit wines made from blackcurrant, crowberry, and bilberry are slightly superior to red wines [27]. In most cases, the contribution of the pigment fraction to the total antioxidant activity of plant phenolic extracts and matrices (i.e., wine and blueberry) was prevalent with respect to the total crude extracts, indicating the high biological and nutraceutical value of these compounds in food matrices [39].

Anthocyanins and flavonols can act as hydroxyl radical (HO^•^) quenchers and strongly inhibit endogenous diffusible free radicals (NO^•^) [44]. Carotenoids react readily with ROS or elevate the body’s own antioxidant defense system through Nrf2 signaling pathways, acting as direct scavengers or quenchers of ROS [66], stopping the propagation of oxidative chain reactions, and inhibiting cell damage [20]. The various molecular configurations of carotenoids are manifested by conjugated double bonds and conjugated double-bond systems that are rich in electrons, which largely influence their antioxidant or pro-oxidant activities [5,18]. Recent trials in term newborns have shown that lutein supplementation lowers systemic oxidative stress [19]. Lutein and zeaxanthin selectively accumulate in human macular cells and act as antioxidants to protect against light-induced macular impairment [66]. Similar to lutein and zeaxanthin, β-cryptoxanthin exhibited antioxidant activity in vitro [19]. β-Carotene and lutein are two of the most prevalent carotenoids in the human diet, and both pigments serve as antioxidants, scavenging free radicals and quenching singlet oxygens [67]. Substantial in vitro work suggests that carotenes are excellent free radical and singlet oxygen quenchers [19]. This unique chemical structure allows chlorophyll to scavenge harmful free radicals and reduce oxidative stress, thereby mitigating DNA damage and preserving the structure and function of neurons, potentially decelerating the initiation or progression of neurodegenerative illnesses [6]. Ferruzi et al. observed that standard chlorophyll a and metallochlorophyll derivatives displayed higher antioxidant capacities than chlorophyll b derivatives [6]. However, it is worth noting that understanding the antioxidant capabilities of natural pigments is a developing field, and ongoing research is crucial to fully understand their potential and establish their importance in comparison to other phytochemicals.

### 5.2. Anti-Inflammation Effects

Anthocyanins and acylated anthocyanins significantly lowered obesity-associated inflammation [68,69]. Carotenoids also play a positive role in the inhibition of inflammation [70,71]. Carotenoids exert anti-inflammatory effects through their systemic anti-inflammatory and antioxidant properties, through the gut microbiota, or by affecting tight junction integrity [63]. On the other hand, carotenoids have the ability to target multiple points along the inflammatory signaling axis, such as the Nrf2 signaling pathway and the NF-κB pathway [22], hence reinstating immune homeostasis and improving the clinical outcomes of inflammatory ailments [20]. Lutein, β-carotene, and lycopene supplementation lowered systemic inflammatory markers in preterm infants [19]. Inflammatory bowel disease (IBD) is strongly related to gut barrier functionality, and β-carotene reduces systemic markers of inflammation and increases occludin expression in the colon [63]. Circulating fibrinogen is an acute-phase protein that is elevated during inflammation, and β-cryptoxanthin levels are inversely associated with circulating fibrinogen [19]. A water extract from the leaves of the red algae Dulse, composed of chlorophyll a and phycobiliproteins, in vitro attenuated the secretion of interleukin-6 (IL-6), proinflammatory tumor necrosis factor-α (TNF-α), and nitric oxide induced by LPS [6].

### 5.3. The Ability to Relieve Metabolic Diseases

Cardiovascular diseases (CVDs) are the principal cause of morbidity and mortality worldwide [39]. Accumulating evidence suggests a positive role of anthocyanins in preserving cardiovascular health [72]. Clinical studies and epidemiological data have shown an association between the consumption of anthocyanins and different anthocyanin-rich sources and a lower risk of surrogate markers of cardiovascular risk, myocardial infarction, and cardiovascular disease-related mortality [72]. A study of 38,180 men and 60,289 women (mean age of 70 and 69 years) with a 7-year follow-up observed a 21% reduction in the risk of CHD mortality and a 14% reduction in CVD mortality in men and women when comparing higher anthocyanin intake (≥16.7 mg/day) with lower intake (<5.5 mg/day) [72]. When compared with controls, a diet containing cyanidin-3-O-β-glucoside (or its aglycone cyanidin) significantly reduces body fat accumulation and plasma glucose concentration induced by high-fat meals [10]. Cyanidin-3-O-β-glucoside also decelerates the atherogenesis caused by diabetes induced in apoE-deficient mice by improving both the loss of endothelial progenitor cell function and endothelial repair [39]. Moreover, the well-characterized anthocyanins from blueberry–blackberry wine blends strongly inhibited DPP-IV and α-glucosidase activities, with anthocyanins from blackberry wine being the most effective at reducing the activity of DPP-IV [73].

Case–control and cohort studies by Bahonar et al. in 2000–2017 revealed that higher administration of the six primary carotenoids (lycopene, lutein, α/β carotene, astaxanthin, and zeaxanthin) was connected to reduced risks of stroke and other cardiovascular events [66]. Lycopene has appeared as a major cardioprotective compound due to its significant antiatherogenic effects, especially in preventing the oxidation of low-density lipoproteins (LDL) [20]. Increased lutein status (intake or serum concentration) was associated with a lower risk of stroke, coronary heart disease, and metabolic syndrome but not with the risk of type 2 diabetes [19]. Low serum β-carotene levels were closely associated with the risk of congestive heart failure, CVD mortality, and sudden cardiac death [19]. New results have also demonstrated the positive effects of carotenoids, including β-carotene, β-cryptoxanthin, fucoxanthin, and astaxanthin, in preventing obesity [7]. Dietary β-carotene addition lowers adiposity in mice in a BCO1-dependent manner. These health benefits are attributed to anti-inflammatory and antioxidant properties [63].

Investigation of the influences of chlorophyll and its derivatives on the digestion of soybean oil in simulated human gastrointestinal environments revealed that chlorophyll reduced the release rate of free fatty acids, changed the fatty acid composition, and increased the particle size of oil droplets [6]. Supplementation with Spirulina extract, a microalga rich in chlorophyll a, prevented the gain of body weight, fat mass, triglyceride, and total cholesterol levels and lowered the expression of adipogenic proteins while increasing thermogenic factors in mice fed an HFD [6]. The anti-adipogenic function of chlorophyll a appears to be mediated through the activation of the AMPK signaling transduction pathway [6].

### 5.4. Other Biological Functions

Recent studies have suggested that anthocyanins have beneficial effects on endothelial function [74]. Anthocyanins may also directly influence gut health via the immune cells found in gut-associated lymphoid tissues [75]. Bilberry anthocyanin consumption (20 mg/kg/day) activated astrocytes and microglia and improved their phagocytic function in APP/PSEN1 mice, thereby ameliorating neurodegenerative diseases [39]. Blueberry anthocyanins exhibit neuroprotective effects by reducing oxidative stress in the eye tissue and preventing the symptoms of diabetic retinopathy [10]. Compared to other anthocyanins, delphinidin 3-rutinoside is particularly potent in relaxing ciliary smooth muscle [10]. Roselle, which is composed of delphinidin-3-O-sambubioside and cyanidin-3-O-sambubioside, ameliorates cardiac function and reduces cardiomyocyte hypertrophy and fibrosis [39]. Results from other cohorts of adults living in the Mediterranean region have shown that moderate alcohol consumption, mainly represented by wine, is associated with a lower risk of depression [76], among which anthocyanidins may play a predominant role [77]. Restricted cubic spline analysis revealed a significant linear relationship between dietary anthocyanidin intake and depression (P for non-linear = 0.5876) [78]. The consumption of anthocyanins showed a significant inverse association with depressive symptoms while reducing depressive symptoms in a dose-dependent manner [76]. Mitochondrial enzymes catalyze the oxidation of monoamines in multiple tissues, and their elevated activity has long been associated with the etiology of depression [76]. Current scientific evidence from animal models suggests a potential role of anthocyanins in suppressing monoamine oxidases, which may explain the antidepressant effects of anthocyanins and their related products [76]. Berry anthocyanins and their aglycones inhibit monoamine oxidase isoforms A and B, with IC(50) values in the low micromolar range achieved by anthocyanidins and anthocyanidin-3-glycosides [79].

Carotenoids inhibit pathogenic growth, improve cognitive function, inhibit cancer, and improve vision. Carotenoids in *Shatian pummelo* peel reduced the growth of potentially pathogenic bacteria, especially *Escherichia coli*, *Bacillus subtilis*, *Staphylococcus aureus*, *Aspergillus niger*, *Aspergillus flavus*, and *Penicillium chrysogenum* [63]. In vitro studies show that β-cryptoxanthin stimulates osteoblastic bone formation and inhibits osteoclastic bone resorption [19]. Several carotenoids may improve the gut barrier function, thus affecting gut health [63]. Carotenoids have also attracted interest as promoters of cognitive function and skin protectants [7]. Recent epidemiological and intervention studies have suggested that dietary and serum lutein and zeaxanthin levels are associated with improved cognitive function during aging and brain function during early life [19]. A high natural carotenoid intake, especially lycopene and β-carotene, improves defense against ultraviolet radiation [20], prevents ultraviolet radiation [25], and decreases sensitivity to UV-induced erythema [66]. β-Carotene has been applied as a photo protector in patients suffering from pathological photosensitivity, including porphyria, although their continuous intake does not show any positive impact on non-melanoma cancer rates relative to a control group [25]. Moreover, diets rich in carotenoids such as lycopene and β-carotene have shown strong protective outcomes against the development and progression of these malignancies [20]. An inverse relationship was found between blood lycopene levels or lycopene intake and the risk and severity of prostate cancer [19]. Both plasma α- and β-carotene were associated with a lower risk of estrogen receptor-negative breast cancer tumors in one recent nested case–control study [19]. Carotenoid intake and blood concentrations of α-carotene, β-carotene, β-cryptoxanthin, total carotenoids, and retinol were all significantly inversely associated with lung cancer risk or mortality among current smokers in a recent meta-analysis and systematic review [19]. Notably, the protective functions of carotenoids, particularly lutein and zeaxanthin in the eye, have been well-demonstrated [22]. Lutein and zeaxanthin preferentially are the exclusive carotenoids that accumulate in the retina and lenses of primate ocular tissue [19]. Lutein and zeaxanthin are collectively known as macular pigments, and dietary lutein and zeaxanthin may help prevent age-related macular degeneration [66]. Lutein has also been linked to a protective effect against eye diseases, such as cataracts and macular degradation [67]. A recent meta-analysis concluded that lutein and zeaxanthin supplementation was inversely related to the risk of advanced age-related macular degeneration and improved contrast sensitivity and visual function in a dose-dependent manner [19]. Interactions with oxidative stress and inflammatory pathways and with NR-mediated signaling might explain many of these effects [7].

Recent investigations have shown that the ability of chlorophyll to modulate oxidative stress and trap mutagens by limiting their bioavailability makes it a good candidate for anticancer activity [80]. Studies have demonstrated the potential anti-carcinogenic action of chlorophyll and sodium copper chlorophyllin against different classes of cancers [6]. In mice, natural chlorophylls reduce colonic cytotoxicity, colonic epithelial cell growth, epithelial cell turnover, and lipid radical production caused by heme, whereas pretreatment with chlorophyll b mitigates chromosomal breakage and micronucleus formation caused by cisplatin in peripheral blood and bone marrow cells [6]. Similar protective effects were observed in the liver and kidneys, where DNA damage was reduced [6]. These studies revealed the potential antigenotoxic and antimutagenic activities of natural chlorophylls [6]. In addition, chlorophyll-rich diets are related to improved cognitive function and brain health [6].

## 6. The Bioavailability of Fruit Wine Pigments

The health benefits and structure–activity relationships of fruit wine pigments are closely related to the bioavailability of pigment molecules, as the biological activities of pigment compounds may be mediated by their metabolites. Pigment compounds generally show low bioavailability when they interact with the food matrix and metabolic processes mediated by the liver (phase I and II metabolism), intestine, and microbiota [81].

After intake, some dietary anthocyanins are rapidly transported to the portal vein, and systemic circulation occurs in the complete glycoside form in the stomach [72]. Anthocyanins are mainly metabolized in the small and large intestines and reach the portal vein circulation through intestinal epithelial cells in the metabolite form [72]. Therefore, only approximately 1–2% maintain their original structure after ingestion, and anthocyanins and their phase II conjugates appear rapidly in circulation [56,66]. They reach a maximal concentration of approximately 100 nM within 1.5 h and disappear from the bloodstream by 6 h post-consumption [72]. Anthocyanins were also found in brain tissue after oral administration [26]. Phenolic acids, including protocatechuic acid, syringic acid, vanillic acid, phloroglucinol aldehyde, phloroglucinol acid, and gallic acid, are chemically derived anthocyanin metabolites [10]. They are thought to be formed during anthocyanin metabolic processing by enteric bacteria or by chemical reactions [10]. The cardiovascular protective effects of anthocyanins are attributed to their metabolites, such as protocatechuic acid [10]. In clinical research on visual function, anthocyanin intake at a daily dose of 50 mg has been shown to confer health benefits [10]. Regarding other physiological functions, there have been few reports on the optimal intake dose for beneficial effects in humans [10]. Additionally, it is important to consider whether non-anthocyanin components affect the effects of anthocyanins alone [10]. Daily food intake is estimated to contain approximately 50 carotenoids that can be absorbed and used in the human body. In the human blood plasma, however, this number is reduced to around 20, from which the major ones are β-carotene, α-carotene, β-cryptoxanthin, lycopene, lutein, and zeaxanthin [7]. The transporter SR-B1 is responsible for the uptake of some carotenoids and is expressed in the intestine; thus, carotenoids may be absorbed in both the small and large intestines and partially transported into the blood circulation system in their original forms [82,83]. Provitamin A carotenoids, including α-carotene, β-carotene, and β-cryptoxanthin, can release retinal by the central cleavage reaction with β-carotene 15,15′-oxygenase within epithelial cells during absorption in the small intestine [66]. Other carotenoids can also produce procarotenoids that enter the circulation. Thus, a wide range of carotenoids of different types and concentrations were confirmed in the primate sera [66]. Very low amounts are passed to the colon because the concentrations of apo-carotenoids within the upper gastrointestinal tract remain relatively low in relation to precursor carotenoids [63]. Some metabolites, such as apo-carotenoids, have biological effects because of higher aqueous solubility and higher electrophilicity that could better target transcription factors, i.e., NF-κB, PPARγ, and RAR/RXRs [63]. For example, C15 (abscisic acid) and C20 apocarotenoids have been shown to improve insulin resistance in a murine model of type 2 diabetes [63]. Among apocarotenoids, an ADI has been established by EFSA for β-apo-8-carotenal at 0.3 mg kg−1 body weight per day [22]. To date, there are no specific recommendations for carotenoid intake [7]. The liver is a major storage site for carotenoids, and a major portion of lycopene resides in adipose tissue [19]. Carotenoid absorption can be relatively low and variable depending on the physicochemical structure, food matrix effects, and a variety of host factors [63]. β-Cryptoxanthin (mainly present in fruits as ester forms) may be the most efficiently absorbed carotenoid [19] and appears to be greater than that of α-carotene or even β-carotene [7]. All-trans β-carotene is more bioavailable than cis isomers [19]. Free lutein shows greater serum lutein responses than lutein ester supplements, while the bioavailability of esterified and free β-cryptoxanthin are comparable [19].

Following the consumption of a single dose of chlorophyll (2–3 mg of 14C pheophytin a), 2.1% of the labeled phytol tail was detected in the human lymph, whereas 90–95% of the labeled phytol tail was detected in the feces [84]. This indicates that approximately 95% of chlorophyll and chlorophyll derivatives were excreted in mammalian feces, with little to no intact chlorophyll being absorbed [84]. Native chlorophyll undergoes significant transformations during digestive processes [6]. Of the fecal excretion products observed, only native chlorophyll a was found in the feces of the mice [6], and most were chlorophyll derivatives, pheophytins, and pyropheophytins, which served as the primary excretion products [84]. The chemical structure of chlorophylls enables their bioactivity [6]. In vitro and in vivo findings regarding native chlorophylls reveal that the potential health benefits related to chlorophylls a and b are attributable to their metal-free derivative compounds [6].

## 7. Overview and Perspectives

We reviewed and discussed the pigment molecules that determine the color of fruit wine from the perspective of biochemical transformation and health benefits. Pigment molecules in fruits undergo a series of dynamic changes during fermentation and aging and eventually become stable in fruit wine. These changes determine the final color of the fruit wine and are affected by a variety of physical, chemical, and microbiological factors. Owing to the action of the pigment itself, its metabolic derivatives in the body, or the influence of matrix effects, fruit wine, and its colored substances exhibit pleiotropic biological functions such as antioxidant and anti-inflammatory effects, inhibition of depression, and improvement of cardiovascular health and vision. Notably, the basis of fruit wine color regulation and pigment molecule characterization involves the matrix balance of the pigment molecules and their derivatives. Physical techniques such as pressure and ultrasound inhibit the activity of enzymes related to pigment oxidation and improve the intensity, brightness, and stability of fruit wine color. Non-covalent interactions between pigments and co-pigmented molecules and complex formation between pigments and water-soluble proteins provide a more stable fruit wine color. With the application of this knowledge, the color, appearance, and sensory quality of fruit wine are expected to be improved during actual fruit wine production.

Although people have learned more about fruit wine color, further in-depth studies in the future on fruit wine color are needed to resolve the various knowledge gaps. These aspects can be listed as follows. (1) Accurate characterization of pigment substances. A large number of pigments in fruit wine have not been characterized, and the properties of polymer pigments and pigment molecules produced by the oxidation of various phenolic substances remain unclear. (2) The application of enzymes. Enzymes are the products of various microorganisms in the fermentation system of fruit wine production. The enzymes produced by genetically modified microorganisms may have great potential for application in fruit wine production within the framework of ensuring absolute safety and legal approval in the future. (3) Clarification of the structure–biological relationship of pigments and their derivatives. It is currently unclear which molecular structures and existing forms of pigments are responsible for specific health benefits, and it is necessary to comprehensively characterize the pigment derivatives generated in the gastrointestinal tract and better understand their pharmacokinetic profiles. Moreover, exploring the synergistic interactions between pigments and other bioactive compounds could offer insights into the holistic benefits of consuming pigment-rich fruit wine.

## Figures and Tables

**Table 1:** Comparison of different physical factors.

Physical Factors	Pigment or Color	Carrier	Change	Reference
Drought	Anthocyanins	Grape skin	Content ↑	[41]
Hot	Carotenoid	Grape	Level ↑	[33]
Cultivar	Carotenoid	Chardonnay wines	β-Damascenone > β-Ionone one	[33]
Time	Ratios of beta-carotene to lutein	Port wines	Aged > New	[32]
	Carotenoid and chlorophyll-like compounds	Port wines	Aged < New	[32]
	Polymer pigments and pyrananthocyanins	Wine	Importance ↑	[27]
	Polymeric pigments	Aged mulberry wines	Formation *↑*	[38]
Humidity	Yellow color	Wine	↑	[42]
Ultrasound	Anthocyanins	Blueberry wine	Stability ↑	[43]
	Anthocyanins	Sanhua plum	Stability ↑	[43]
	Color	Aged mulberry wine	Intensity ↑	[38]
	Total anthocyanin	Wine	Content ↑	[44]
	Dark	Mulberry wine	↑	[38]
Maceration	Most glycosidic pigments and anthocyanins	Bayberry wine	Content ↓	[1]
Pressure	Color	Mulberry wine	Stability ↑	[38]
	Monomeric anthocyanins and flavonols	Mulberry wine	Content ↓	[38]
	Yellow pigments	Mulberry wine	Color tonality ↑	[38]
	Bright	Mulberry wine	↑	[38]
Sunlight	Carotenoid	Ripe grape	Concentrations ↓	[33]
Red light	β-Cryptoxanthin	Fruit wine	Content ↑	[15]
Manosonication	Blue tone	Mulberry wine	↓	[38]
	Chlorophyll	Fruit wine	Chl b > Chl a	[45]

↑: Increase; ↓: Decrease.

**Table 2:** Comparison of different chemical factors.

Chemical Factors	Pigment or Color	Carrier	Change	Reference
Acidity	Anthocyanins	Fruit wine	Stability ↑ Red ↑	[30,36,46,53]
Alkalinity	Anthocyanins	Fruit wine	Content ↓ Blue ↑	[36,39,46]
O_2_	Chlorophylls and carotenoids	Fruit wine	Content ↓	[21]
	Browning	Fruit wine	↑	[28]
	Anthocyanin	Fruit wine	Bluish–red ↓ Yellow–orange ↑	[27]
CO_2_	Chlorophyll, total carotenoids, 9-cis-violaxanthin	Fruit wine	Content ↓	[15]
	Orange-colored β-cryptoxanthin	Fruit wine	Content ↑	[15]
Co-pigment	Purple hue	Fruit wine	↑	[34]
	Anthocyanins	Fruit wine	Red color ↑	[38]
Hydroxylation	Yellow hue	Fruit wine	↑	[49]
	Anthocyanidin	Fruit wine	Stability ↓ Blue ↑ Red ↓	[11,49]
Methylation	Red color	Fruit wine	↓	[11]
Cleavage	Carotenoid	Wine	Degradation ↑ Color ↓	[21,33]
Soluble sugar	Color	Wine	↓	[50]
	Pyranoanthocyanins	Wine	Content ↑	[51,52]
Fining agents (chitosan, gelatin, and agar)	Color	Citrus wine	Lightens ↑	[36]

↑: Increase; ↓: Decrease.

**Table 3:** Comparison between different microbiological factors.

Microbiological Factors	Pigment or Color	Carrier	Change	Reference
Yeast	Pigment molecules	Fruit wine	Transformation ↑	[48,49]
Mold	Anthocyanin	Fruit wine	Consumption ↓	[48,51]
	Pigment molecules	Fruit wine	Stability ↑	[56]
Bacteria	Anthocyanin	Fruit wine	Consumption ↓	[48,51]
Mannoprotein	Anthocyanin	Blueberry wine	Stability ↑	[34]
	Delphinidin	Blueberry wine	Blue ↑	[34]

↑: Increase; ↓: Decrease.

## Data Availability

No new data were created or analyzed in this study. Data sharing is not applicable to this article.

## References

[1] Merken H.M., Beecher G.R. (2000). Measurement of Food Flavonoids by High-Performance Liquid Chromatography: A Review. J. Agric. Food Chem..

[2] Blanco-Gomis D., Mangas-Alonso J.J., Junco-Corujedo S., Gutiérrez-Álvarez M.D. (2007). Cider Proteins and Foam Characteristics:  A Contribution to Their Characterization. J. Agric. Food Chem..

[3] Pando Bedriñana R., Rodríguez Madrera R., Picinelli Lobo A., Mérillon J.-M., Rivière C., Lefèvre G. (2025). Production of New Ciders: Chemical and Sensory Profiles. Natural Products in Beverages.

[4] Zhang X., Li X., Fang H., Guo F., Li F., Chen A., Huang S. (2019). Flavonoids as Inducers of White Adipose Tissue Browning and Thermogenesis: Signalling Pathways and Molecular Triggers. Nutr. Metab..

[5] Kertész K., Piszter G., Horváth Z.E., Bálint Z., Biró L.P. (2017). Changes in Structural and Pigmentary Colours in Response to Cold Stress in *Polyommatus icarus* Butterflies. Sci. Rep..

[6] Martins T., Barros A.N., Rosa E., Antunes L. (2023). Enhancing Health Benefits through Chlorophylls and Chlorophyll-Rich Agro-Food: A Comprehensive Review. Molecules.

[7] Rodriguez-Concepcion M., Avalos J., Bonet M.L., Boronat A., Gomez-Gomez L., Hornero-Mendez D., Limon M.C., Meléndez-Martínez A.J., Olmedilla-Alonso B., Palou A. (2018). A Global Perspective on Carotenoids: Metabolism, Biotechnology, and Benefits for Nutrition and Health. Prog. Lipid Res..

[8] Albert N.W., Lewis D.H., Zhang H., Irving L.J., Jameson P.E., Davies K.M. (2009). Light-Induced Vegetative Anthocyanin Pigmentation in *Petunia*. J. Exp. Bot..

[9] Ali H.M., Ali I.H. (2018). Energetic and Electronic Computation of the Two-Hydrogen Atom Donation Process in Catecholic and Non-Catecholic Anthocyanidins. Food Chem..

[10] Tsuda T. (2012). Dietary Anthocyanin-Rich Plants: Biochemical Basis and Recent Progress in Health Benefits Studies. Mol. Nutr. Food Res..

[11] Tanaka Y., Brugliera F., Chandler S. (2009). Recent Progress of Flower Colour Modification by Biotechnology. Int. J. Mol. Sci..

[12] Ferrándiz M.L., Devesa I. (2008). Inducers of Heme Oxygenase-1. Curr. Pharm. Des..

[13] Cataldo E., Fucile M., Manzi D., Masini C.M., Doni S., Mattii G.B. (2023). Sustainable Soil Management: Effects of Clinoptilolite and Organic Compost Soil Application on Eco-Physiology, Quercitin, and Hydroxylated, Methoxylated Anthocyanins on *Vitis vinifera*. Plants.

[14] Li Y., Chen S., Lyu X., Fang X., Cao X. (2024). Metabolomic Analysis to Unravel the Composition and Dynamic Variations of Anthocyanins in Bayberry-Soaked Wine during the Maceration Process. Food Chem. X.

[15] Lu Y., Li D., Li L., Belwal T., Xu Y., Lin X., Duan Z., Luo Z. (2020). Effects of Elevated CO_2_ on Pigment Metabolism of Postharvest Mandarin Fruit for Degreening. Food Chem..

[16] Li Y., Huang X., Luo L., Shang C. (2022). Optimization of Extraction Conditions of Carotenoids from *Dunaliella parva* by Response Surface Methodology. Molecules.

[17] Abdel-Aal E.-S.M., Mats L., Rabalski I. (2022). Identification of Carotenoids in Hairless Canary Seed and the Effect of Baking on Their Composition in Bread and Muffin Products. Molecules.

[18] Naithani R., Huma L.C., Moriarty R.M., McCormick D.L., Mehta R.G. (2008). Comprehensive Review of Cancer Chemopreventive Agents Evaluated in Experimental Carcinogenesis Models and Clinical Trials. Curr. Med. Chem..

[19] Moran N.E., Mohn E.S., Hason N., Erdman J.W., Johnson E.J. (2018). Intrinsic and Extrinsic Factors Impacting Absorption, Metabolism, and Health Effects of Dietary Carotenoids. Adv. Nutr..

[20] Bas T.G. (2024). Bioactivity and Bioavailability of Carotenoids Applied in Human Health: Technological Advances and Innovation. Int. J. Mol. Sci..

[21] Gutiérrez-Gamboa G., Marín-San Román S., Jofré V., Rubio-Bretón P., Pérez-Álvarez E.P., Garde-Cerdán T. (2018). Effects on Chlorophyll and Carotenoid Contents in Different Grape Varieties (*Vitis vinifera* L.) after Nitrogen and Elicitor Foliar Applications to the Vineyard. Food Chem..

[22] Meléndez-Martínez A.J. (2019). An Overview of Carotenoids, Apocarotenoids, and Vitamin A in Agro-Food, Nutrition, Health, and Disease. Mol. Nutr. Food Res..

[23] Ishibashi M., Zaitsu K., Yoshikawa I., Otagaki S., Matsumoto S., Oikawa A., Shiratake K. (2023). High-Throughput Analysis of Anthocyanins in Horticultural Crops Using Probe Electrospray Ionization Tandem Mass Spectrometry (PESI/MS/MS). Hortic. Res..

[24] Sollazzo M., Baccelloni S., D’Onofrio C., Bellincontro A. (2018). Combining Color Chart, Colorimetric Measurement and Chemical Compounds for Postharvest Quality of White Wine Grapes. J. Sci. Food Agric..

[25] Bosch R., Philips N., Suárez-Pérez J.A., Juarranz A., Devmurari A., Chalensouk-Khaosaat J., González S. (2015). Mechanisms of Photoaging and Cutaneous Photocarcinogenesis, and Photoprotective Strategies with Phytochemicals. Antioxidants.

[26] Barbalho S.M., Bueno Ottoboni A.M.M., Fiorini A.M.R., Guiguer É.L., Nicolau C.C.T., Goulart R.d.A., Flato U.A.P. (2020). Grape Juice or Wine: Which Is the Best Option?. Crit. Rev. Food Sci..

[27] Kalkan Yildirim H. (2006). Evaluation of Colour Parameters and Antioxidant Activities of Fruit Wines. Int. J. Food Sci. Nutr..

[28] Zhang X.-K., Jeffery D.W., Li D.-M., Lan Y., Zhao X., Duan C.-Q. (2022). Red Wine Coloration: A Review of Pigmented Molecules, Reactions, and Applications. Compr. Rev. Food Sci. Food Saf..

[29] Venter A., Joubert E., de Beer D. (2014). Nutraceutical Value of Yellow- and Red-Fleshed South African Plums (*Prunus salicina* Lindl.): Evaluation of Total Antioxidant Capacity and Phenolic Composition. Molecules.

[30] Sánchez R., González M.R., Fernández-Fernández E., Rodríguez-Nogales J.M., Martín P. (2020). Relationships between Chlorophyll Content of Vine Leaves, Predawn Leaf Water Potential at Veraison, and Chemical and Sensory Attributes of Wine. J. Sci. Food Agr..

[31] Teixeira A., Noronha H., Frusciante S., Diretto G., Gerós H. (2023). Biosynthesis of Chlorophyll and Other Isoprenoids in the Plastid of Red Grape Berry Skins. J. Agric. Food Chem..

[32] Mendes-Pinto M.M., Silva Ferreira A.C., Caris-Veyrat C., Guedes de Pinho P. (2005). Carotenoid, Chlorophyll, and Chlorophyll-Derived Compounds in Grapes and Port Wines. J. Agric. Food Chem..

[33] Crupi P., Coletta A., Milella R.A., Palmisano G., Baiano A., La Notte E., Antonacci D. (2010). Carotenoid and Chlorophyll-Derived Compounds in Some Wine Grapes Grown in Apulian Region. J. Food Sci..

[34] Sun X., Yan Z., Zhu T., Zhu J., Wang Y., Li B., Meng X. (2019). Effects on the Color, Taste, and Anthocyanins Stability of Blueberry Wine by Different Contents of Mannoprotein. Food Chem..

[35] Li Q., Chang X.-X., Wang H., Brennan C.S., Guo X.-B. (2019). Phytochemicals Accumulation in Sanhua Plum (*Prunus salicina* L.) during Fruit Development and Their Potential Use as Antioxidants. J. Agric. Food Chem..

[36] Bi J., Li H., Wang H. (2019). Delayed Bitterness of Citrus Wine Is Removed Through the Selection of Fining Agents and Fining Optimization. Front. Chem..

[37] Yao X.-C., Zhang H.-L., Ma X.-R., Xia N.-Y., Duan C.-Q., Yang W.-M., Pan Q.-H. (2024). Leaching and Evolution of Anthocyanins and Aroma Compounds during Cabernet Sauvignon Wine Fermentation with Whole-Process Skin-Seed Contact. Food Chem..

[38] Tchabo W., Ma Y., Kwaw E., Zhang H., Xiao L., Apaliya M.T. (2018). Statistical Interpretation of Chromatic Indicators in Correlation to Phytochemical Profile of a Sulfur Dioxide-Free Mulberry (*Morus nigra*) Wine Submitted to Non-Thermal Maturation Processes. Food Chem..

[39] Mattioli R., Francioso A., Mosca L., Silva P. (2020). Anthocyanins: A Comprehensive Review of Their Chemical Properties and Health Effects on Cardiovascular and Neurodegenerative Diseases. Molecules.

[40] Urvieta R., Buscema F., Bottini R., Coste B., Fontana A. (2018). Phenolic and Sensory Profiles Discriminate Geographical Indications for Malbec Wines from Different Regions of Mendoza, Argentina. Food Chem..

[41] Daccak D., Lidon F.C., Luís I.C., Marques A.C., Coelho A.R.F., Pessoa C.C., Caleiro J., Ramalho J.C., Leitão A.E., Silva M.J. (2022). Zinc Biofortification in *Vitis vinifera*: Implications for Quality and Wine Production. Plants.

[42] Tamayo-Sánchez J.C., Meza-González D.A., Warren-Vega W.M., Zárate-Guzmán A.I., Romero-Cano L.A. (2023). Advances in the Development of Tailor-Made Color Alcoholic Beverages Based on an Accelerated Maturation Process. Food Res. Int..

[43] Wu Z., Li X., Zeng Y., Cai D., Teng Z., Wu Q., Sun J., Bai W. (2022). Color Stability Enhancement and Antioxidation Improvement of Sanhua Plum Wine under Circulating Ultrasound. Foods.

[44] Tchabo W., Ma Y., Kwaw E., Zhang H., Li X., Afoakwah N.A. (2017). Effects of Ultrasound, High Pressure, and Manosonication Processes on Phenolic Profile and Antioxidant Properties of a Sulfur Dioxide-Free Mulberry (*Morus nigra*) Wine. Food Bioprocess Technol..

[45] Solymosi K., Mysliwa-Kurdziel B. (2017). Chlorophylls and Their Derivatives Used in Food Industry and Medicine. Mini. Rev. Med. Chem..

[46] Yang X., Sun H., Tu L., Jin Y., Zhang Z., Wang M., Liu S., Wang Y., He S. (2020). Kinetics of Enzymatic Synthesis of Cyanidin-3-Glucoside Lauryl Ester and Its Physicochemical Property and Proliferative Effect on Intestinal Probiotics. Biology.

[47] Ogodo A.C., Ugbogu O.C., Ugbogu A.E., Ezeonu C.S. (2015). Production of Mixed Fruit (Pawpaw, Banana and Watermelon) Wine Using *Saccharomyces cerevisiae* Isolated from Palm Wine. SpringerPlus.

[48] Montero R., Mundy D., Albright A., Grose C., Trought M.C.T., Cohen D., Chooi K.M., MacDiarmid R., Flexas J., Bota J. (2016). Effects of *Grapevine Leafroll* Associated Virus 3 (GLRaV-3) and Duration of Infection on Fruit Composition and Wine Chemical Profile of *Vitis vinifera* L. Cv. Sauvignon Blanc. Food Chem..

[49] Cheng S., Wu T., Gao J., Han X., Huang W., You Y., Zhan J. (2023). Color Myth: Anthocyanins Reactions and Enological Approaches Achieving Their Stabilization in the Aging Process of Red Wine. Food Innov. Adv..

[50] Alcalde-Eon C., Pérez-Mestre C., Ferreras-Charro R., Rivero F.J., Heredia F.J., Escribano-Bailón M.T. (2019). Addition of Mannoproteins and/or Seeds during Winemaking and Their Effects on Pigment Composition and Color Stability. J. Agric. Food Chem..

[51] Ji J., Henschen C.W., Nguyen T.H., Ma L., Waterhouse A.L. (2020). Yeasts Induce Acetaldehyde Production in Wine Micro-Oxygenation Treatments. J. Agric. Food Chem..

[52] Wang S., Li S., Zhao H., Gu P., Chen Y., Zhang B., Zhu B. (2018). Acetaldehyde Released by *Lactobacillus plantarum* Enhances Accumulation of Pyranoanthocyanins in Wine during Malolactic Fermentation. Food Res. Int..

[53] Zheng T., Zhang D.-L., Sun B.-Y., Liu S.-M. (2022). Evaluating the Impacts of Climate Factors and Flavonoids Content on Chinese Prickly Ash Peel Color Based on HPLC-MS and Structural Equation Model. Foods.

[54] Tempère S., Marchal A., Barbe J.-C., Bely M., Masneuf-Pomarede I., Marullo P., Albertin W. (2018). The Complexity of Wine: Clarifying the Role of Microorganisms. Appl. Microbiol. Biotechnol..

[55] Imre A., Kovács R., Tóth Z., Majoros L., Benkő Z., Pfliegler W.P., Pócsi I. (2022). Heme Oxygenase-1 (HMX1) Loss of Function Increases the In-Host Fitness of the *Saccharomyces ‘boulardii’* Probiotic Yeast in a Mouse Fungemia Model. J. Fungi.

[56] Jiang L., Qiu Y., Dumlao M.C., Donald W.A., Steel C.C., Schmidtke L.M. (2023). Detection and Prediction of *Botrytis cinerea* Infection Levels in Wine Grapes Using Volatile Analysis. Food Chem..

[57] Cortell J.M., Halbleib M., Gallagher A.V., Righetti T.L., Kennedy J.A. (2005). Influence of Vine Vigor on Grape (*Vitis vinifera* L. Cv. Pinot Noir) and Wine Proanthocyanidins. J. Agric. Food Chem..

[58] Martín-Tornero E., de Jorge Páscoa R.N.M., Espinosa-Mansilla A., Martín-Merás I.D., Lopes J.A. (2020). Comparative Quantification of Chlorophyll and Polyphenol Levels in Grapevine Leaves Sampled from Different Geographical Locations. Sci. Rep..

[59] Agati G., D’Onofrio C., Ducci E., Cuzzola A., Remorini D., Tuccio L., Lazzini F., Mattii G. (2013). Potential of a Multiparametric Optical Sensor for Determining in Situ the Maturity Components of Red and White *Vitis vinifera* Wine Grapes. J. Agric. Food Chem..

[60] Yang Z., Tian J., Feng K., Gong X., Liu J. (2021). Application of a Hyperspectral Imaging System to Quantify Leaf-Scale Chlorophyll, Nitrogen and Chlorophyll Fluorescence Parameters in Grapevine. Plant Physiol. Biochem..

[61] Taylor K.L., Brackenridge A.E., Vivier M.A., Oberholster A. (2006). High-Performance Liquid Chromatography Profiling of the Major Carotenoids in Arabidopsis Thaliana Leaf Tissue. J. Chromatogr. A.

[62] Lombardo M., Feraco A., Camajani E., Caprio M., Armani A. (2023). Health Effects of Red Wine Consumption: A Narrative Review of an Issue That Still Deserves Debate. Nutrients.

[63] Eroglu A., Al’Abri I.S., Kopec R.E., Crook N., Bohn T. (2022). Carotenoids and Their Health Benefits as Derived via Their Interactions with Gut Microbiota. Adv. Nutr..

[64] Čakar U., Čolović M., Milenković D., Pagnacco M., Maksimović J., Krstić D., Đorđević B. (2025). Strawberry and Drupe Fruit Wines Antioxidant Activity and Protective Effect Against Induced Oxidative Stress in Rat Synaptosomes. Antioxidants.

[65] Xu A., Xiao Y., He Z., Liu J., Wang Y., Gao B., Chang J., Zhu D. (2022). Use of Non-*Saccharomyces* Yeast Co-Fermentation with *Saccharomyces cerevisiae* to Improve the Polyphenol and Volatile Aroma Compound Contents in Nanfeng Tangerine Wines. J. Fungi.

[66] Terao J. (2023). Revisiting Carotenoids as Dietary Antioxidants for Human Health and Disease Prevention. Food Funct..

[67] Maia M., Cavaco A.R., Laureano G., Cunha J., Eiras-Dias J., Matos A.R., Duarte B., Figueiredo A. (2021). More than Just Wine: The Nutritional Benefits of Grapevine Leaves. Foods.

[68] Jayarathne S., Stull A.J., Park O.-H., Kim J.H., Thompson L., Moustaid-Moussa N. (2019). Protective Effects of Anthocyanins in Obesity-Associated Inflammation and Changes in Gut Microbiome. Mol. Nutr. Food Res..

[69] Jokioja J., Yang B., Linderborg K.M. (2021). Acylated Anthocyanins: A Review on Their Bioavailability and Effects on Postprandial Carbohydrate Metabolism and Inflammation. Compr. Rev. Food Sci. Food Saf..

[70] Ruta L.L., Farcasanu I.C. (2019). Anthocyanins and Anthocyanin-Derived Products in Yeast-Fermented Beverages. Antioxidants.

[71] Devi A., Konerira Aiyappaa A., Waterhouse A.L. (2020). Adsorption and Biotransformation of Anthocyanin Glucosides and Quercetin Glycosides by *Oenococcus Oeni* and *Lactobacillus plantarum* in Model Wine Solution. J. Sci. Food Agric..

[72] Krga I., Milenkovic D. (2019). Anthocyanins: From Sources and Bioavailability to Cardiovascular-Health Benefits and Molecular Mechanisms of Action. J. Agric. Food Chem..

[73] Fan J., Johnson M.H., Lila M.A., Yousef G., de Mejia E.G. (2013). Berry and Citrus Phenolic Compounds Inhibit Dipeptidyl Peptidase IV: Implications in Diabetes Management. Evid.-Based Complement. Altern. Med..

[74] Landberg R., Naidoo N., van Dam R.M. (2012). Diet and Endothelial Function: From Individual Components to Dietary Patterns. Curr. Opin. Lipidol..

[75] Blesso C.N. (2019). Dietary Anthocyanins and Human Health. Nutrients.

[76] Godos J., Castellano S., Ray S., Grosso G., Galvano F. (2018). Dietary Polyphenol Intake and Depression: Results from the Mediterranean Healthy Eating, Lifestyle and Aging (MEAL) Study. Molecules.

[77] Deng M.-G., Liu F., Wang K., Zhang M.-J., Feng Q., Liu J. (2024). Association between Dietary Flavonoid Intake and Depressive Symptoms: A Cross-Sectional Research. Gen. Hosp. Psychiatry.

[78] Chen W., Zhao J. (2023). Association between Dietary Anthocyanidins Intake and Depression among US Adults: A Cross-Sectional Study (NHANES, 2007–2010 and 2017–2018). BMC Psychiatry.

[79] Dreiseitel A., Korte G., Schreier P., Oehme A., Locher S., Domani M., Hajak G., Sand P.G. (2009). Berry Anthocyanins and Their Aglycons Inhibit Monoamine Oxidases A and B. Pharmacol. Res..

[80] Agarry I.E., Ding D., Li Y., Jin Z., Deng H., Hu J., Cai T., Kan J., Chen K. (2023). In Vitro Bioaccessibility Evaluation of Chlorophyll Pigments in Single and Binary Carriers. Food Chem..

[81] Di Lorenzo C., Colombo F., Biella S., Stockley C., Restani P. (2021). Polyphenols and Human Health: The Role of Bioavailability. Nutrients.

[82] Zhao H., Liu S., Zhu L., Wang Y. (2025). Microorganisms: The Key Regulators of Wine Quality. Comp. Rev. Food Sci. Food Safe..

[83] Sternes P.R., Lee D., Kutyna D.R., Borneman A.R. (2017). A Combined Meta-Barcoding and Shotgun Metagenomic Analysis of Spontaneous Wine Fermentation. Gigascience.

[84] Zhong S., Bird A., Kopec R.E. (2021). The Metabolism and Potential Bioactivity of Chlorophyll and Metallo-Chlorophyll Derivatives in the Gastrointestinal Tract. Mol. Nutr. Food Res..

